# Cross-cultural validation of the decisional balance scale in exercise across countries

**DOI:** 10.1371/journal.pone.0257511

**Published:** 2021-09-30

**Authors:** Yee Cheng Kueh, Abdulwali Sabo, Youngho Kim, Garry Kuan

**Affiliations:** 1 Biostatistics and Research Methodology Unit, School of Medical Sciences, Universiti Sains Malaysia, Kelantan, Malaysia; 2 Department of Community Medicine, Federal University Dutse, Dutse, Jigawa State, Nigeria; 3 Department of Sport Science, Seoul National University of Science and Technology, Seoul, South Korea; 4 Exercise and Sports Science, School of Health Sciences, Universiti Sains Malaysia, Kelantan, Malaysia; 5 Department of Life Sciences, Brunel University London, Uxbridge, United Kingdom; UCSI University, MALAYSIA

## Abstract

**Background:**

This study examined the psychometric properties of the Korean and Malay version of the decisional balance (DB) for exercise (i.e. perceived benefits and perceived barriers) using a cross-sectional design. Also, this study assessed the measurement and structural invariance of the DB scale across countries.

**Methods:**

A cross sectional study was conducted in Malaysia and Korea. The study sample consisted of 574 Korean participants and 562 Malaysian participants. The mean age of the participants was 19.8 (SD = 1.29) for the Korean sample and 19.8 (SD = 1.22) for the Malaysian sample. Participants were invited to complete the DB scale with the 10-item and two factors (i.e., perceived benefit and perceived barriers). Confirmatory factor analysis (CFA) and invariance test were conducted on the data by using Mplus 8.3.

**Results:**

The CFA results based on the hypothesised measurement model of two factors and ten items showed sufficient construct validity after adding residual covariance between items within the same factor: CFI = 0.979, TLI = 0.970, SRMR = 0.036, RMSEA = 0.036 for the Korea sample, and CFI = 0.964, TLI = 0.949, SRMR = 0.055, RMSEA = 0.066 for the Malay sample. For the Korea sample, the construct reliability was 0.62 and 0.74 for perceived benefits and perceived barriers respectively. For the Malay sample, the construct reliability was 0.75 and 0.77 for perceived benefits and perceived barriers respectively. The findings presented evidence for measurement and structural invariance of the DB scale for the Korea and Malaysia samples.

**Conclusion:**

The DB scale was a valid and reliable measure for assessing exercise behaviour and for making comparisons between Korean and Malaysian samples.

## Introduction

It has been well reported that regular physical activity can significantly improve quality of life and reduce the risk of heart disease and obesity [1]. Physical activity behaviours acquired during childhood may progress into adulthood [2]. Despite the numerous benefits of physical activity, it remains the fourth leading risk factor for global mortality, causing about 3.2 million deaths annually [3]. In Malaysia, 39.7% of the population spent most of their time in sedentary activities [4], and a Korean study reported that only 8–9% of the subjects were physically active [5].

Over the years, the Transtheoretical Model (TTM) has been recognised as one of the most popular theoretical frameworks employed for physical activity-based interventions [6, 7]. The TTM, originally developed by Prochaska [8], is an integrative psychological framework of behavioural change that aims to increase theoretical diversity within one’s health behaviour choice and motivation. The TTM model has been used in developing interventions that promote behavioural change circumstances such as substance abuse, diet, and exercise [2, 9].

Interventions meant to promote physical activity behaviour must consider physical activity as a complex behaviour to understand the extent of willingness and human variations between different phases [10]. The TTM framework has a dynamic approach to understand different aspects of physical activity behaviour, such as different stages of readiness [11]. Given that, many studies have reported the success of the TMM in promoting adherence and maintenance of exercise behaviours [2, 10, 12, 13]. The TTM is a multidimensional model that involves five key constructs, i.e. stages of change, the process of change, decisional balance, self-efficacy and temptation [14]. This study focuses on the decisional balance components of the TTM.

The decisional balance construct is an important component of the struggle model that is needed when making decision-related on specific health behaviour [15]. Decisional balance reflects the perceived benefits (pros) and perceived barriers (cons) of varying exercise behaviour. These two factors combine to determine the relative potential gains and losses of exercise behaviour. The pros increase, while the cons decrease across the stages of change for exercise behaviour (i.e. pre-contemplation to maintenance) [16]. As individuals progress to making healthy exercise behaviour, the values of their cons gradually shift from cons to pros [2]. This study focuses on confirming the psychometric properties of the Malay and Korean versions of the decisional balance scale and its factorial invariance across the Malay and Korean population.

The Decisional Balance (DB) scale was originated by Marcus et al. [17], reflecting two factors: (1) perceived benefits (pros, ten items) concerning the positive aspects of exercise behaviour and (2) the perceived barriers (cons, six items) concerning the negative aspects of exercise behaviour. The DB scale was reviewed and revised among 543 Canadian adults (18–65 years old) pilot study with two factors and 10 items [18], and tested the measurement invariance of their revised 10-item DB scale across time using a random sample of 703 Canadian adults, ageing between 18 to 65 years old. Their results revealed that the revised DB scale with 10 items and two factors remained invariant across time (12-month period) with a Cronbach alpha coefficient ranging from.77 to.83 for the perceived benefit factor and from.69 to.72 for the perceived barrier factor. Brown et al. [19], supported the use of the revised DB scale by Plotnikoff et al. [18], to determine structural relationships between DB constructs and other constructs of interest. The validity and reliability of the DB scale constructs and their structural relationship with other psychological constructs were confirmed by previous researchers [20, 21]. Additionally, the DB scale was translated and confirmed to have adequate construct validity in Malay [22] and Korean [21] using CFA.

The structural invariance was ascertained across time using 10-items and two factors. The factorial validity of the Malay decisional balance scale was also ascertained across gender, making it a valid scale for making a comparison between male and female [22]. However, given the complex nature of physical activity behaviour and its diversity across different cultures [10, 23], in the present study, the researchers further assessed the factorial validity of the DB scale across different cultures (i.e., Malaysia and Korea) using the Malay DB scale [22] and Korean DB scale [21]. Thus, the first study to determine the ability of the DB scale when making a comparison between different countries. As such, no translation process was performed in this study since the researchers used the translated scales by previous researchers [21, 22].

There is a prevailing high interest in understanding and promoting physical activity behaviour and how it is associated and influenced by several psychological factors such as the decisional balance, with evidence that allows the comparisons of the decisional balance constructs between different factors of interest. As such, it is necessary to establish a measurement scale with adequate psychometric properties that will precisely reflect the constructs of the decisional balance scale. Physical activity behaviour is influenced by various factors such as cultural values, community, age, gender, family, and peers [23]. These multitudes of biological and sociocultural variables need attention across human lifespan in order to determine appropriate physical activity behaviour [23]. Given that, cultural variations influence an individual’s exercise behaviour, in this study, we aimed to confirm its factorial invariance across Malaysian and Korean students.

## Methods

### Participants

For testing measurement invariance, the minimum recommended sample size is *n* = 200 per group [24]. However, a simulation study by Meade and Lautenschlager [25], revealed that a large sample size of 500 and 1000 produce a better result for testing measurement equivalence of covariance matrices and factor loadings across different groups. In the present study, the researchers considered a sample size of 500 for each group as adequate. A 20% dropout rate was added to account for missing data and the new adjusted sample size was 625. Therefore, a total of 625 questionnaires were distributed to each Malaysian and Korean university students. The total of 574 questionnaires and 562 questionnaires with complete responses from Korean and Malaysian students, respectively, were returned to the researchers and used in this study. The overall mean and standard deviation (SD) for age were 19.8 years old (SD = 1.26) and majority were female (79%).

### Measures

The DB scale for physical activity was a self-reported measure to assess perceived benefits and barriers of exercise behaviours evaluated with the 5-point response options ordering from 1 (not at all important) to 5 (very much). The translated Malay version by Kuan et al. [22], and translated Korean version by Lee and Kim [21], were applied in this study. An example of the perceived benefits item is “Physical activity would help me reduce tension or manage stress” while an example of the perceived barriers item is “Physical activity would take too much of my time”. Previous studies reported the two weeks test-retest reliability of 0.98 (perceived benefits) and 0.96 (perceived barriers) for the Malay DB scale [22], and 0.83 (perceived benefits) and 0.80 (perceived barriers) for the Korean DB scale [21].

### Procedure

A cross-sectional study was conducted in a university located in Malaysia and Korea. The study was approved by the Human Research Ethics Committee, Universiti Sains Malaysia and Seoul National University of Science and Technology. Participation in this study by completing the questionnaire was voluntary and based on written consent. Convenience sampling was used in recruiting the participants to answer the questionnaire. The data collection was carried out at the end of the student’s lectures and tutorials. The students were briefed regarding the purpose of the study and how to complete the questionnaires, and only those who volunteered to participate in the study were given the study questionnaires. Hence, the study participants were those present and signed written consent to participate during the data collection and able to read and understand the language of the questionnaires. The participants took about 10–15 minutes to complete the questionnaire which consisted of some demographic information and DB scale.

### Data analysis

Data analysis was conducted using Mplus 8.3 [26]. There were no missing data in the dataset. The assumption of multivariate normality was not met based on Mardia multivariate skewness and kurtosis tests of fit (*p* < 0.001). Therefore, an alternative estimator robust to maximum likelihood, MLM was used in the confirmatory factor analysis (CFA) and measurement of invariance testing.

The hypothesised measurement model consists of two latent variables (factors) and 10 observed variables (items). The factor structure of the hypothesised measurement model was first tested in CFA separately for Korea and Malaysia samples. The goodness of fit indices used in assessing the measurement model were: the comparative fit index (CFI) and the Tucker and Lewis index (TLI), with the desired value of more than 0.95, the root mean square error of approximation (RMSEA), with the desired value of less than 0.08, and the Standardised Root Mean Residual (SRMR), with the desired value of less than 0.08 [27]. The Chi-square and its degree of freedom (df) were reported along with other fit indices. Factor loadings (with recommended value of above 0.40), standardised residuals, and modification indices (MI), were used to identify any problematic items that contributed to misfit to the data [28]. Additional parameters, such as residual covariances among items, were added in the re-specification models based on MI values. Model was re-specified after consideration of meaningfulness of adding the covariances among the identified items. The construct reliability of the best fit measurement model of both countries was computed following Raykov’s method [29] in Mplus 8.3. The recommended value for construct reliability is equal or above 0.60 [27].

Measurement and structural invariances across the countries was tested based on published guidelines by Byrne [30]. In this hierarchical tests, a less restrictive model was first identified, and this model was called configural model. In this model, same number of factors and the factor loading pattern were imposed across the groups and no equality constraints are imposed on any of the parameters. Next, equivalence of the factor loadings across the countries were examined. A more restrictive model was identified with all factor loading were constrained to be equaled across the countries. The common residual covariances are not constrained equal by default in Mplus. Therefore, further constraint was imposed on the common residual covariances of both countries.

In this model the factor loadings are treated invariant across the two countries. This ensures that the measures are considered to be on the same scale across the countries for making valid comparisons. Third, we examined the strong invariance model. This model is invariance on both factor loadings and item intercept across the two countries. This is to ensure the underlying factors can be compared across countries. Fourth, we examined the strict invariance model, which requires the factor loadings, intercepts, and residual variances to be invariant. This is to examine whether the variances of the regression equations for each item are invariant across countries. After testing the invariance related to measurement model, the next is to test the invariance of structural parameters in the model which include only the factor variances and covariances. At this stage, a more restrictive model was identified, equality constraints were imposed on factor loadings, residual covariances, the factor variances and covariances.

Evidence of invariance between the less restrictive model and more restrictive model were based on recommendations from the literature [28, 31–33]. A value of the change in CFI (ΔCFI) smaller than or equal to 0.01 indicates that the hypothesis of invariance should not be rejected. For ΔRMSEA, the critical value is 0.015. The Chi-square difference test was also reported for each comparison.

## Results

### Participant characteristics

A total 574 Korean and 562 Malaysian completed the Decisional Balance scale. The mean age (in year) of the participants were almost similar for both groups, 19.8 (*SD* = 1.29) for the Korean sample and 19.8 (*SD* = 1.22) for the Malaysian sample. In the present study, the female consisted of 79% for both the Malay and Korean samples, while, the male consisted of 21% for both the Malaysian and Korean samples. For the Korean sample, the mean of perceived benefits and perceived barriers were 3.83 (*SD* = 0.72) and 2.27 (*SD* = 0.82) respectively. For the Malay sample, the mean of perceived benefits and perceived barriers were 3.82 (*SD* = 0.72) and 2.27 (*SD* = 0.83) respectively.

### Measurement model of decisional balance scale

The hypothesized measurement model (hypothesised model) for Decisional Balance scale consists of two factors and 10 items. The baseline model (Korea model-1) for Korea sample did not fit the data well. Model re-specification was conducted by including two residual covariances (items 1 & 2, items 4 & 5). The re-specified model for Korea sample fit the data well based on several fit indices (see Table 2, Korea Model-2).

Similar to Korea sample, the baseline model (Malaysia model-1) for Malaysia sample did not fit the data well. Model re-specification was done by including two residual covariances (items 1 & 2, items 6 & 7) and the re-specified model reflect a satisfactorily good fit to the data based on several fit indices (see Table 2, Malaysia Model-2).

**Fig 1:**
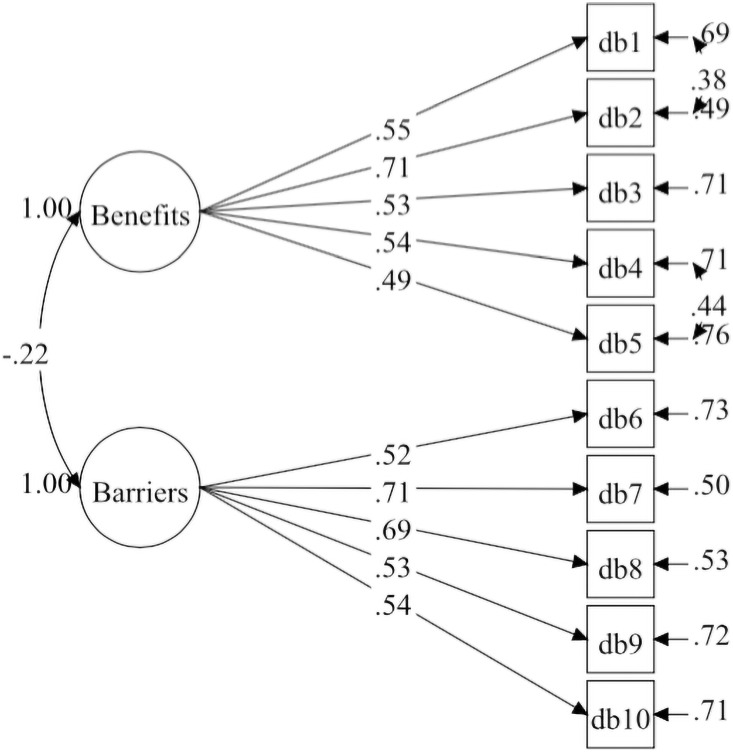
Decisional balance measurement model for Korea-based sample (model-2).

**Fig 2:**
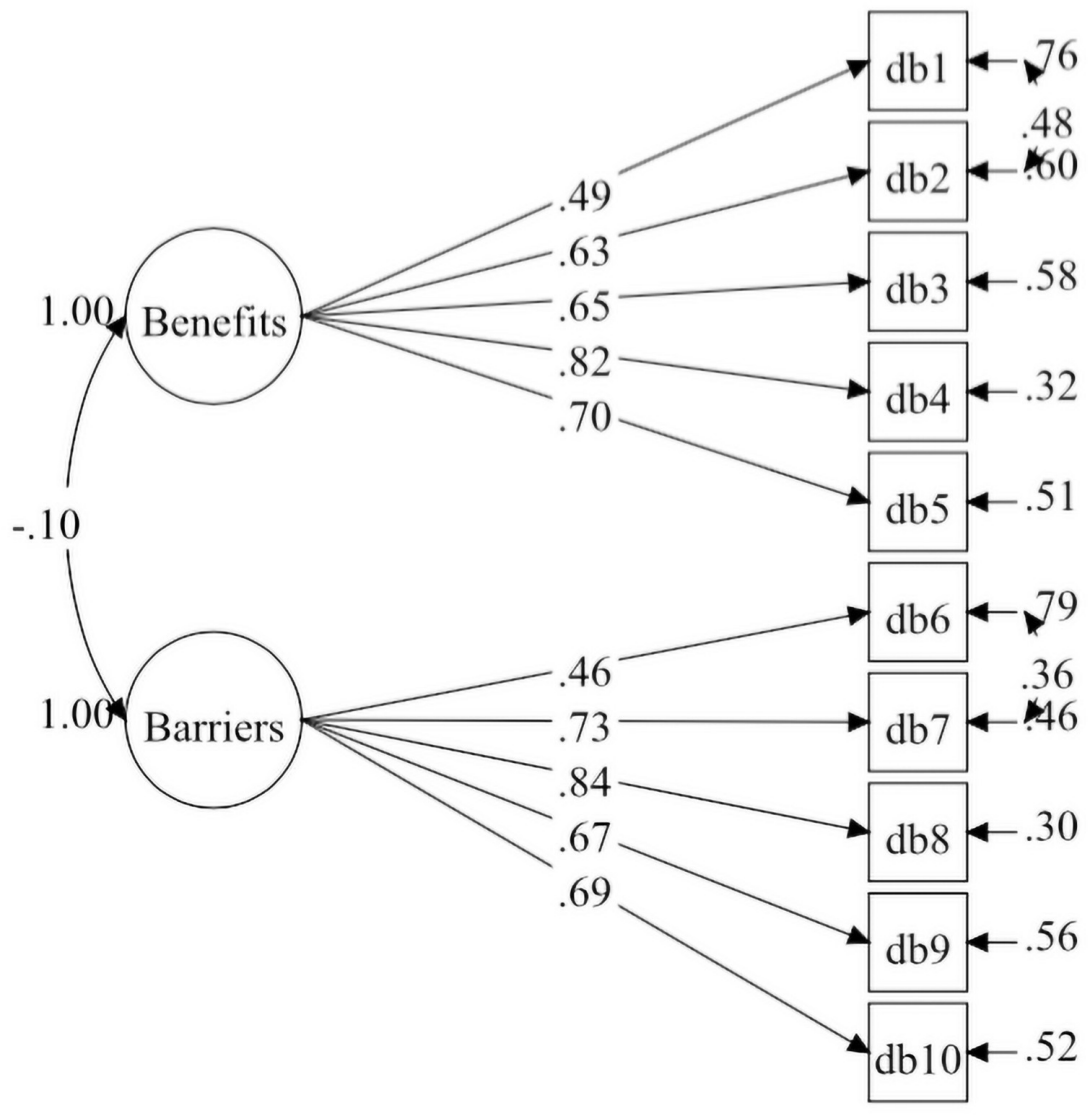
Decisional balance measurement model for Malaysia-based sample (model-2).

Both Korea Model-2 and Malaysia Model-2 were similar but not completely identical. The standardised factor loadings for each factor within the Korea and Malaysia Models-2 are illustrated in Table 1. The construct reliability based on Korea Model-2 and Malaysia Model-2 were satisfactory and ranged from 0.62 to 0.77. The Korea Model-2 and Malaysia Model-2 are illustrated in a CFA diagram in Figs 1 and 2.

**Table 1:** Standardised factor loadings for Korea Model-2 and Malaysia Model-2.

Factors	Items	Standardized factor loading		Construct reliability	
		Korea sample^b^	Malaysia sample^c^	Korea sample	Malaysia sample
Benefit	Item 1	0.55	0.49	0.62	0.75
	Item 2	0.71	0.63		
	Item 3	0.53	0.65		
	Item 4	0.54	0.82		
	Item 5	0.49	0.70		
Barrier	Item 6	0.52	0.46	0.74	0.77
	Item 7	0.71	0.73		
	Item 8	0.69	0.84		
	Item 9	0.53	0.67		
	Item 10	0.54	0.69		

*Note*.

^a^Adding residual covariances between items 1 & 2, and 4 & 5.

^b^Adding residual covariances between items 1 & 2 and 6 & 7

### Measurement and structural invariance

The two baseline models (Korea and Malaysia samples) had the same two factors and we found that all 10 items fell into their hypothesized factors. These two baseline models were then integrated into the configural invariance model, with the same number of factors and the same pattern of fixed and free factor loadings, but no equality constraint was imposed on any parameter across the Malay and Korean samples. The configural invariance model fit the data well (see Table 2). This configural model was then used to compare against the more restrictive measurement invariance and structural invariance.

**Table 2:** Baseline model fit results and tests of measurement invariance.

Models	MLM χ^2^, (df)	CFI	TLI	RMSEA	SRMR	Model comparison	ΔMLM χ^2^ *, (df), p-value	ΔCFI	ΔTLI	ΔRMSEA
Korea Model-1 (hypothesized)	190.161 (34)	0.861	0.816	0.090	0.056	-	-	-	-	-
Korea Model-2 (respecified)^a^	55.636 (32)	0.979	0.970	0.036	0.036	-	-	-	-	-
Malaysia Model-1 (hypothesized)	239.353 (34)	0.885	0.848	0.104	0.063	-	-	-	-	-
Malaysia Model-2 (respecified)^b^	96.739 (32)	0.964	0.949	0.060	0.055	-	-	-	-	-
Model-3 (configural model)	152.135 (64)	0.970	0.957	0.049	0.046	-	-	-	-	-
Measurement invariance:						-	-	-	-	-
Model-4 (all factor loading invariant)	176.150 (72)	0.964	0.955	0.051	0.054	4 versus 3	20.798 (8), 0.008	0.006	0.002	0.002
Model-5 (all factor loading invariant, one common residual covariance invariant)	179.568 (73)	0.963	0.955	0.051	0.054	5 versus 4	3.140 (1), 0.076	0.001	0	0
Structural invariance:										
Model-6 all factor loading invariant, one common residual covariance invariant, factor variance and covariances invariant)	215.388 (76)	0.952	0.943	0.057	0085	6 versus 5	35.749 (3), <0.001	0.011	0.012	0.006

Note.

*Corrected value.

^a^Adding residual covariances between item 1 & 2 and 4 & 5,

^b^Adding residual covariances between items 1 & 2 and 6 & 7, Δ = change in value

The first more restrictive model (Model-4), the measurement invariance with all factor loading invariant, fit the data well (see Table 2). Changes of CFI, TLI and RMSEA, when the Model-4 is compared with the Model-3 (configural model), were within acceptable values (ΔCFI = 0.006, ΔTLI = 0.002, ΔRMSEA = 0.002). This indicates that the metric of factor scores was invariant across the two countries. In other words, the items used to estimate the factor loadings have the same meaning for Korean and Malaysian students. The next restrictive model, the measurement invariance with all factor loadings invariant and one common residual covariance invariant (Model-5) also fit the data well (see Table 2). When comparing Model-5 (more restricted) and Model-4 (less restricted), changes of CFI, TLI and RMSEA were small and within acceptable values (ΔCFI = 0.001, ΔTLI = 0, ΔRMSEA = 0). This indicates that factor loadings and the common residual covariance are invariant between the two countries. The structural invariance for the factor variances and covariances model fit the data well based on several fit indices (see Table 2). The differences of several fit indices with the less restrictive invariance model (i.e., Model-5) are within the acceptable values for RMSEA (ΔRMSEA = 0.006). However, the changes in CFI and TLI were slightly over the recommended value (ΔCFI = 0.011, ΔTLI = 0.012).

## Discussion

The present study aimed to evaluate the construct validity and reliability of the Malay and Korean versions of the DB scales using CFA. Also, the researcher assessed the measurement and structural invariance of the DB scale across Malaysia and the Korean sample. This study presented evidence for adequate psychometric properties of the DB-M scale consistent with previous studies [21, 22]. The DB scales fit the data well with all the ten items retained. The factor loadings were adequate (above 0.40), ranging from 0.50–0.71 and 0.46–0.84 for the Korean and Malay DB scales respectively. Furthermore, the results presented solid evidence for the measurement and structural invariance across the countries.

It is further relevant to note that the aim of the present study, was to examine the measurement and structural invariance of the DB scale across the different cultures of Malaysia and Korea using the translated Malay version and translated Korean version. However, to achieve that, it is necessary to determine the psychometric properties of these scales. The findings of the present study present evidence of the DB scale psychometric properties which supported the revised 10-items DB scale by [18]. All the psychometric fit indices of the final models were substantial with desired items loadings on their respective factors. These are also in line with the psychometric studies of the 10-items DB scale by previous researchers [20, 22, 34–36].

The decisional balance is a psychological aspect of the TTM that influence exercise behaviour [2, 20, 21]. According to the TTM, several attempts are required to make behavioural change successful [2]. Hence, it is necessary to evaluate the reliability of the decisional balance constructs. Both the Malay and Korean DB scales showed sufficient construct validity. The construct reliabilities exceeded the prescribed value of.60 [37]. This result revealed that both the DB scales had sufficient construct validity, and all the items accurately measure their respective factors. Also, a previous study by Kuan et al. [22], reported a construct reliability of 0.85 for perceived benefits and 0.84 for perceived barriers.

In the present study, we examined the measurement invariance of the DB scale across countries. The findings revealed evidence for adequate measurement invariance [28, 38]. These indicate that the Korean and Malaysian samples interpreted all the ten items of the DB scale similarly. Hence, a valid comparison of the decisional balance for exercise behaviour can be made across these countries. Furthermore, the structural invariance of the DB scale was desirable based on RMSEA cutoff points but slightly higher based on CFI and TLI cutoff points across the countries. These results indicate that the relationships between the factors and the means of the factors may slightly vary across the countries. This is consistent with the previous study by Seefeldt et al. [23], that community and cultural values have an influence on exercise behaviour. There was no previous study that reported the measurement and structural invariance of the DB scale across countries. Nonetheless, previous studies revealed that the DB scale has adequate measurement invariance across gender [22] and across time [18].

In the present study, the residual covariances between items within the same construct were added to obtain desired fit indices of the model. These residual covariances were added based on the MI values reported in the Mplus output. Also, the researchers considered the theoretical meaning of these residual covariances. For the Korea sample, Covariance between residuals for items DB1 (Physical activity would help me reduce tension or manage stress) and DB2 (I would feel more confident about my health by getting physical activity), and between DB4 (Physical activity would help me have a more positive outlook) and DB5 (Physical activity would help me control my weight) were added. For the Malay sample, Covariance between residuals for items DB1 (Physical activity would help me reduce tension or manage stress) and DB2 (I would feel more confident about my health by getting physical activity), and between DB6 (I am too tired to get physical activity because of my other daily responsibilities) and DB7 (Physical activity would take too much of my time) were added. These residual covariances indicate shared sources of variability between items of the same factor over and beyond the factors [35]. Also, in social and psychological research, these residual covariances can be included when there is a genuine meaning [39, 40].

This study is not without limitations. Firstly, the study is a cross-sectional design, therefore, causal interpretation must be made with caution. Secondly, the data was collected from a single university both in Malaysia and Korea which may affect the generalizability of the study findings. However, the large sample size used in the present study may add more strength to the study findings. Lastly, self-reported measures were used to assess the study outcomes which could lead to response bias and reduce the accuracy of the data collected. Nevertheless, the participants were ensured of their confidentiality and encouraged to respond sincerely to all the items.

More importantly, this study presents evidence of psychometric properties of the 10-items DB scale for assessing perceived benefits and barriers of physical activity behaviour on a population from different countries. Future studies are needed to confirm the external validity of the DB scale using a more robust sampling approach, and re-assess the replicability of the DB scale with a more diverse Korean and Malaysian population of different ages, education levels, occupations, and health conditions. Also, more scale development and validation work are needed to identify more items to enhance the properties of the DB constructs.

## Conclusion

In this study, the DB scale was confirmed to have valid psychometric properties for assessing the decisional balance for exercise behaviour in Malaysian and Korean sample. All the items have desired factor loadings on their respective factor. Also, the scale revealed adequate measurement invariance (configural, weak, strong, and strict) and structural invariance across countries. These findings indicate that the DB scale can be used for making valid comparisons of exercise behaviour across both countries.

## Supporting information

S1 Text (TXT)
